# Endoscopic capsule robot-based diagnosis, navigation and localization in the gastrointestinal tract

**DOI:** 10.3389/frobt.2022.896028

**Published:** 2022-09-02

**Authors:** Mark Hanscom, David R. Cave

**Affiliations:** University of Massachusetts Medical School, Worcester, MA, United States

**Keywords:** capsule, capsule endoscopy, gastrointestinal tract, capsule locomotion, artificial intelligence, AI

## Abstract

The proliferation of video capsule endoscopy (VCE) would not have been possible without continued technological improvements in imaging and locomotion. Advancements in imaging include both software and hardware improvements but perhaps the greatest software advancement in imaging comes in the form of artificial intelligence (AI). Current research into AI in VCE includes the diagnosis of tumors, gastrointestinal bleeding, Crohn’s disease, and celiac disease. Other advancements have focused on the improvement of both camera technologies and alternative forms of imaging. Comparatively, advancements in locomotion have just started to approach clinical use and include onboard controlled locomotion, which involves miniaturizing a motor to incorporate into the video capsule, and externally controlled locomotion, which involves using an outside power source to maneuver the capsule itself. Advancements in locomotion hold promise to remove one of the major disadvantages of VCE, namely, its inability to obtain targeted diagnoses. Active capsule control could in turn unlock additional diagnostic and therapeutic potential, such as the ability to obtain targeted tissue biopsies or drug delivery. With both advancements in imaging and locomotion has come a corresponding need to be better able to process generated images and localize the capsule’s position within the gastrointestinal tract. Technological advancements in computation performance have led to improvements in image compression and transfer, as well as advancements in sensor detection and alternative methods of capsule localization. Together, these advancements have led to the expansion of VCE across a number of indications, including the evaluation of esophageal and colon pathologies including esophagitis, esophageal varices, Crohn’s disease, and polyps after incomplete colonoscopy. Current research has also suggested a role for VCE in acute gastrointestinal bleeding throughout the gastrointestinal tract, as well as in urgent settings such as the emergency department, and in resource-constrained settings, such as during the COVID-19 pandemic. VCE has solidified its role in the evaluation of small bowel bleeding and earned an important place in the practicing gastroenterologist’s armamentarium. In the next few decades, further improvements in imaging and locomotion promise to open up even more clinical roles for the video capsule as a tool for non-invasive diagnosis of lumenal gastrointestinal pathologies.

## Introduction

**FIGURE 1:**
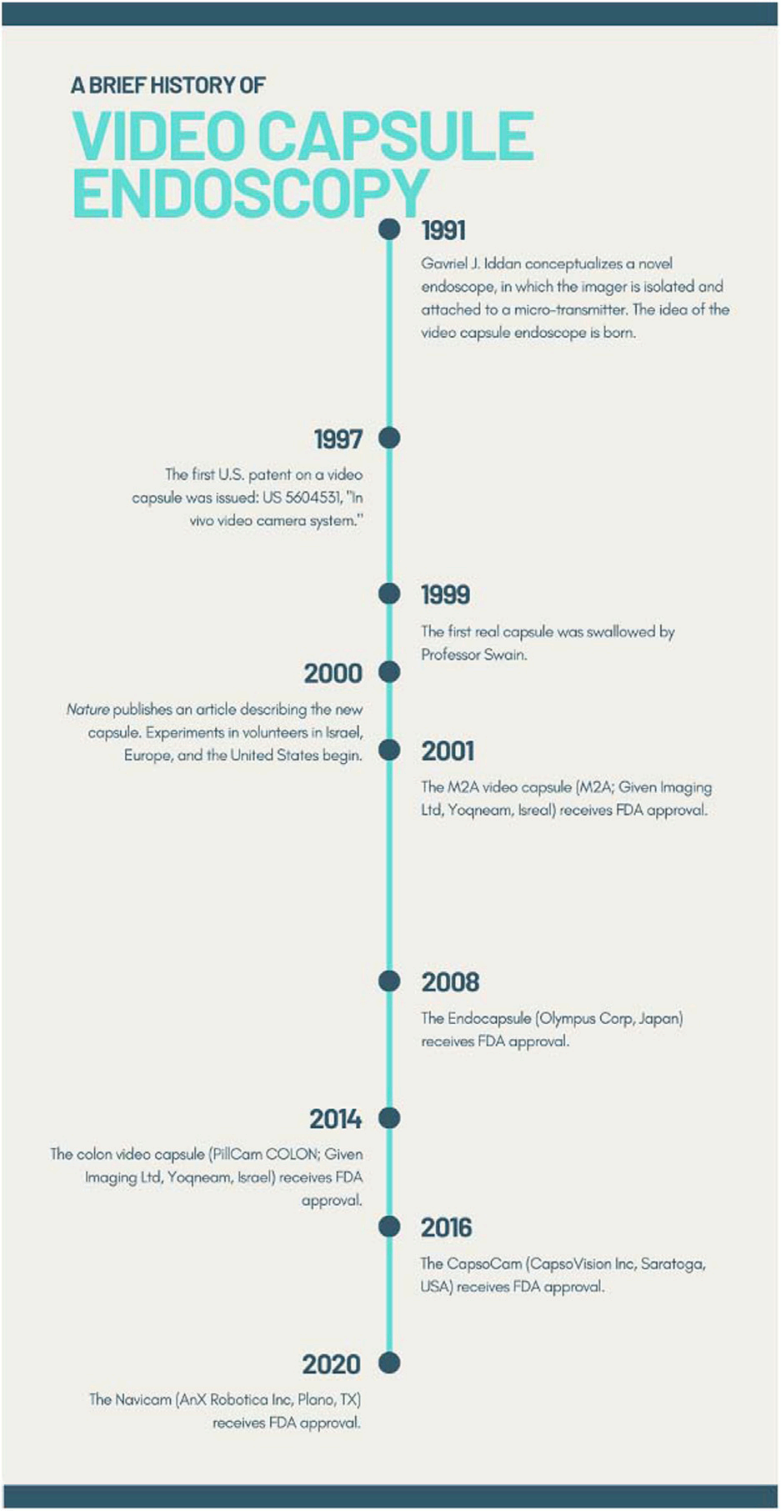
A timeline of important developments in the history of video capsule endoscopy.

The concept of using miniaturized non-invasive cameras to examine the small intestine is now more than 20 years old (Figure 1). (Keuchel et al., 2014) From that initial concept has emerged the field of video capsule endoscopy (VCE), fulfilling the promise of non-invasive imaging of the gastrointestinal (GI) tract and in doing so introducing the practicing gastroenterologist to a new diagnostic tool. Now, 20 years after its conception, VCE has revolutionized the evaluation of the GI tract. VCE has been deployed worldwide for a plethora of different conditions including the detection of bleeding, diagnosis of Crohn’s disease, and detection of tumors. For suspected small bowel bleeding, VCE has become the first-line investigative method. (Gerson et al., 2015).

Yet since its inception, VCE has proffered the potential to explore more than just the small intestine. In its most fundamental form, a video capsule consists of a camera and a vehicle with which to transport it. Indeed, the non-invasive nature of VCE, combined with its ease-of-use and favorable adverse event profile, makes it a promising tool for evaluation of all number of GI pathologies. Despite these advantages, the use of VCE outside of the small intestine has remained underdeveloped. One of the major obstacles to more widespread adoption of VCE has been its reliance on passive locomotion through peristalsis, preventing targeted exams of areas of interest. Related limitations include its inability to perform tissue biopsies or execute therapeutic maneuvers, such as hemostasis or drug delivery. Lastly, the time and attention required to review collected capsule images, in particular in comparison to its better reimbursed counterpart in conventional endoscopy, remains an impediment to more extensive adoption. (Keuchel et al., 2014). However, in the past decade, newer research has expanded what’s possible with VCE and led to its increased proliferation across the field of gastroenterology. These technological advancements are multitude and include upgrades in imaging, locomotion, and localization. In total, these advancements have solidified the role of VCE in existing indications and promulgated its role in newer ones including in the evaluation of acute gastrointestinal bleeding (GIB), gastroesophageal reflux disease (GERD) and non-cardiac chest pain (NCCP), and cirrhosis and portal hypertensive bleeding. In addition, VCE has demonstrated a role in resource-constrained and high-risk settings. In the near future, VCE will become a diagnostic tool for the entire GI tract.

The future of VCE is bright. Technological advancements in imaging, locomotion and localization have spurred the expansion of VCE a great deal since its first introduction to clinical practice in 2001. In the future, continued upgrades in imaging and locomotion are poised to expand its influence even further. Below, we discuss some of these newer adjunctive technologies such as artificial intelligence (AI), machine learning, and internal and externally controlled locomotion, and how they have shaped the developing applications of VCE.

## Advancements in diagnosis

Since its introduction to clinical practice, tissue diagnosis has been one of major limitations of VCE. Current commercial capsules are not engineered for tissue acquisition. To do so would require an additional component—a therapeutic module—designed to obtain tissue. (Keuchel et al., 2014). Recent research into imaging and locomotion has teased some solutions. The first is optical biopsy, which relies on advancements in imaging to establish a diagnosis based on optical parameters alone. The second is the more traditional tissue diagnosis which, while still in nascent stages of research, leverages advancements in locomotion and overall capsule construction to integrate sampling methods into the capsule endoscope. (Scott and Enns, 2015; Slawinski et al., 2015; Hakimian et al., 2021a).

### Advancements in imaging

Advancements in imaging have come in the form of hardware improvements and software improvements, with advancements in engineering spurring numerous upgrades to the various components of the capsule including the camera, power source, and transmission unit. Software improvements, likewise, have paralleled engineering advancements. However, unlike the multitude of improvements on the hardware end, software improvements have in large part focused on the advent of AI and convolutional neural networks (CNN).

### Hardware improvements

Hardware improvements have occurred across each component of the endoscopic capsule, which in its most basic form comprises a camera and image sensor, illumination unit, power source, and transmission unit.

Current commercial video capsules differ in technical specifications depending on their indication (Table 1). For example, several of the small bowel capsules are 26-mm in length x 11-mm in diameter (PillCam SB3, Medtronic Minneapolis, MN, and EndoCapsule, Olympus, Westborough, MA). In comparison, their counterpart intended for colonic imaging (PillCam COLON2) measures 31.5-mm in length x 11-mm in diameter. The weight of most commercial capsules ranges from 1.9-g to 6-g. (Keuchel et al., 2014). Despite these differences in technical specifications, the diagnostic yield between different capsule platforms remains similar with overall agreement approaching 90%. (Pioche et al., 2011; Dolak et al., 2012; Choi et al., 2013). Consequently, the decision of which capsule to use is one based on local resources.

**TABLE 1:** Comparison of technical specifications between different video capsule endoscopy platforms.

Platform	Manufacturer	Cameras	LEDs	Battery life (h)	Frames per second	Field of view (degrees)
PillCam SB3	Medtronic	1	4	>8	2–6	156
PillCam UGI	Medtronic	2	8 (4 per side)	1.5	18–35	172
PillCam COLON 2	Medtronic	2	8 (4 per side)	>10	4–35	172
EndoCapsule	Olympus	1	4	12	2	160
CapsoCam Plus	CapsoVision	4	16	15	20	360
MiroCam	IntroMedic	1	6	11	3	170
OMOM	Jinshan	1	4	12	2–10	172

Most commercial capsules contain a single camera, such as the PillCam SB3 (Medtronic, Minneapolis, MN) and Olympus EC-10 (Olympus Corp, Westborough, MA). However, both two-camera (PillCam UGI and PillCam COLON 2) and four-camera (CapsoCam) capsules have been developed. (Keuchel et al., 2014). Cameras are contained on one or both ends of the capsule with the exception of the CapsoCam (CapsoVision Inc., Saratoga, CA) which contains four radially oriented cameras at its center. (Slawinski et al., 2015). In one small trial of a two-camera capsule in 41 patients, the additional camera was found to complement the first camera and detected 68 positive findings compared to 48 findings with the single camera capsule alone. (Triantafyllou et al., 2011). Despite the potential for improved lesion detection with additional cameras, obstacles to adoption include the need for more powerful batteries and more patient endoscopists, who are in turn tasked with the time and attention-intensive feat of interpreting multiple video streams. The frame rates range from 2 to 20 frames per second. (Keuchel et al., 2014).

The viewing angle of commercial capsules ranges from 145° to 160°. Exceptions include the CapsoCam, which contains four cameras with a 360 degree viewing angle, and the PillCam ESO 2 and PillCam COLON2, which each contain two end mounted cameras at 169° and 172°, respectively. Current capsules have a fixed focal length and depth of focus in the range of 0–50 mm. Several researchers have proposed a “liquid-lens solution,” in which a deformable liquid lens is used in place of a mechanical lens. The liquid lens enables power-efficient extended depth of focus and zoom features. (Keuchel et al., 2014). Other researchers have proposed a side-viewing lens to better map the entire GI lumen although neither innovation has made it into commercial capsules. (Koulaouzidis and Dabos, 2013).

Battery life has been progressively extended over the years. Most capsules now have a life of 12 or more hours, with a range between 8 and 12 h for small bowel video capsules. This enhancement, from about 8 h, has all but eliminated incomplete transit through the small intestine.

The illumination unit consists of four to six light-emitting diodes (LEDs) that emit a flashing white light that serves as the illumination source for the camera. Some studies have experimented with mixing non-white-light (NWL) LEDs at various intensities to simulate white light while reducing power consumption. Outside of power preservation, the use of NWL has also demonstrated potential in lesion detection. The principle behind the use of NWL is that certain tissues, such as pre-malignant or malignant tissues, are predisposed to respond to certain wavelengths of light different from those of normal mucosa. For instance, in flexible spectral imaging color enhancement (FICE), monochromatic wavelengths of light are emphasized in the red, green, and blue spectrums to produce an amalgamated image that better highlights lumenal lesions. Initial studies of FICE reported improved visualization of small bowel angioectasias, erosions, ulcers, and tumors in the range of 20%–87%. However, later blinded studies found a significant difference in the detection of angioectasias alone. (Imagawa et al., 2011; Yung et al., 2017a). The RAPID 6.0 video CE workstation (Given Imaging Ltd., Yokneam, Israel) includes the option for FICE, but clinical usefulness remains controversial. (Gupta et al., 2011; Ibrahim and Van Gossum, 2013). There might be a role for FICE in improving lesion detection in a background of luminal bile pigment. (Sakai et al., 2012).

Narrow band imaging (NBI), which is another form of NWL, has also been applied to VCE in limited context. In NBI, wavelengths in the visible spectrum of light are filtered except for “narrow bands” in the 415–540 nm range, corresponding with the blue and green spectrum. Blood vessels appear more prominent under this filter because the range of light corresponds with the peak absorption of oxygenated hemoglobin. Thus, superficial pit patterns and the associated lesions can be better delineated. Dung et al. first proposed the concept of a NBIcapsule endoscope in 2010, which operated for 6–8 h with a frame rate of 2 frames per second. (Lan-Rong and Yin-Yi, 2010). To date, the capsule has not made it into commercial use.

Blue mode, a subset of NBI in which a filter of light in the blue wavelength is superimposed on to a white-light image, has, however, been incorporated into VCE with mixed results. While blue filtering is thought to enhance mucosal details, in a small review of 27 patients it was not found to improve the overall detection of small bowel inflammation. (Koulaouzidis et al., 2012).

### Artificial intelligence and machine learning

The greatest software improvement has come with the introduction of AI. Convolutional neural networks (CNN) can now process thousands of images to create predictive algorithms that can in turn generate a diagnosis. VCE is well-suited to the advent of AI given its inherent image generation and routine process of image acquisition.

Technical specifications, including CNN architecture, differ between studies. For example, the datasets used for program training range from 300 to well over 100,000. (Trasolini and Byrne, 2021). Programs built on larger datasets would seem more robust but require corresponding amounts of effort and processing power in the form of technical requirements and computation performance to label and learn from each image. Deep learning as well as machine learning programs require these large datasets of information on which to “train,” but in turn demand increased computing power.

The potential benefit of robust AI applied to VCE is enormous as several of the greatest challenges in VCE could be solved or at least ameliorated with an accurate machine learning platform. For example, VCE suffers from long reading times and comparatively low reimbursement, which have been barriers to widespread adoption. Current VCE exams produce over 50,000 images and require between 30 and 120 min to read. (Wang et al., 2013). In addition, pathologies encountered during VCE are numerous and often fleeting. In the near future, AI could reduce much of the time and attention-consuming process of rote reading of capsule endoscopy while also standardizing lesion detection. AI will also assist in the detection of fleeting lesions seen in 1 or 2 frames, which could be missed by a tired reader. To date, the use of AI in VCE has been studied in the detection of GIB, inflammatory bowel disease (IBD), gastrointestinal tumors, and celiac disease with promising results (Tables 2, 3).

**TABLE 2:** Selected studies evaluating the testing characteristics of CNNs in VCE. PPV, NPV, and overall accuracy are included where reported.

Author(s)	Published	Indication	Sensitivity (%)	Specificity (%)	PPV	NPV	Accuracy (%)
Aoki	2019	Erosions and ulcerations	88.2	90.9			90.8
Klang	2019	Crohn’s disease	92.5–97.1	96–98.1	94.4–97.2	94.8–97.9	95.4–96.7
Leenhardt	2019	Angioectasia	100	96	96	100	
Tsuboi	2020	Angioectasia	98.8	98.4	75.4	99.9	
Saito	2020	Protruding lesions	90.7	79.8			98.6
Aoki	2020	GIB	96.6	99.9			99.9

**TABLE 3:** Comparison of the role of AI in various diseases.

Disease	Selected studies	Sensitivity (%)	Advantages	Disadvantages
Erosions and ulcers	Aoki (2019)	88.2	Reduce reading time, image whole bowel	Cannot biopsy, cannot perform hemostasis
Crohn’s disease	Klang (2019)	97.1	Reduce reading time, image whole bowel	Cannot biopsy
Angioectasia	Leenhardt (2019), Tsuboi (2020)	98.8	Expedite triage, reduce low-yield procedures	Cannot biopsy, cannot perform hemostasis
Protruding lesions	Saito (2020)	90.7	Reduce reading time, image whole bowel	Cannot biopsy
Gastrointestinal bleeding	Aoki (2020)	96.6	Expedite triage, reduce low-yield procedures	Cannot biopsy, cannot perform hemostasis
Celiac disease	Zhou (2017)	100	Reduce reading time, non-invasive diagnosis	Cannot biopsy
All abnormalities	Ding (2019)	99.8	Reduce reading time, image whole bowel	Cannot biopsy

**FIGURE 2:**
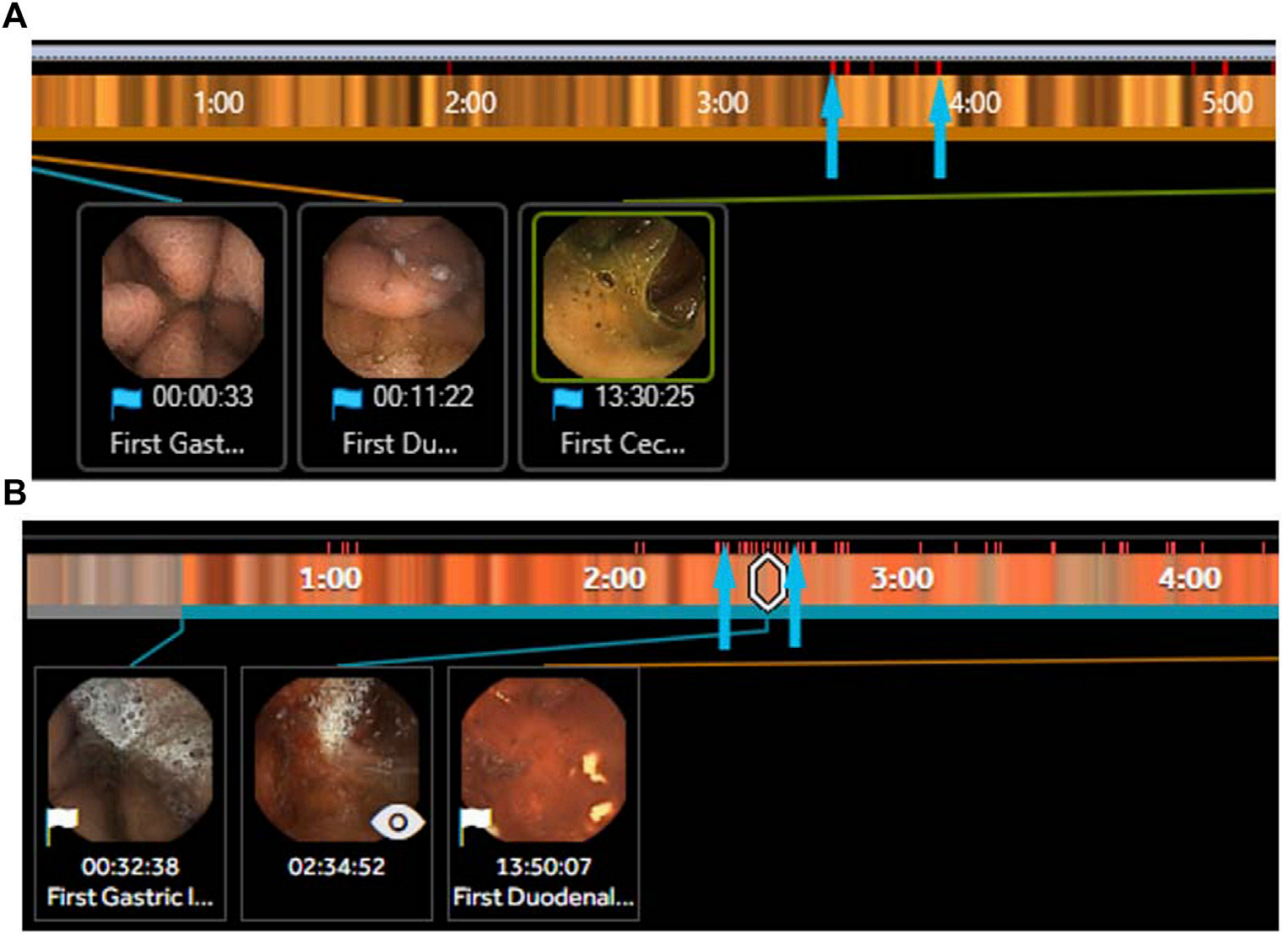
**(A)** Section of the suspected blood indicator (SBI) PillCam SB3 (Medtronic, MN) with several red pixels (arrows) identifying areas of suspected bleeding but are false positives. **(B)** Section of the SBI PillCam SB3 (Medtronic, MN) with a long segment of >8 contiguous red pixels identifying areas of suspected bleeding that are true positives with active bleeding identified in the image inset.

One of the earliest applications of AI in VCE has been the suspected blood indicator (SBI) function (Given Imaging, Israel). The SBI identifies specific frames with a threshold number of red-color pixels and then marks those frames with a distinct red line along the capsule’s timeline (Figures 2A,B). In one of the first studies evaluating the testing characteristics of the SBI, Liangpunsakul et al. (2003) reviewed data from 24 patients who underwent VCE for either iron-deficiency anemia (IDA) or abdominal pain and found an overall sensitivity, specificity, and accuracy of the SBI to detect small bowel lesions of 25.7%, 90%, and 34.8%, respectively). Several more studies have evaluated the role of the SBI for various indications with suboptimal results (Table 4). In one of the largest studies, over 250 patients were evaluated using SBI for a multitude of indications including anemia, obscure GIB, and Crohn’s disease. The sensitivity, specificity, positive predictive value (PPV), and negative predictive value (NPV) for all lesions was 56.4%, 33.5%, 24.0%, and 67.3%, respectively. (Buscaglia et al., 2008). In a subgroup analysis, detection of actively bleeding lesions was only minimally better with a sensitivity and PPV between 58.3% and 70%. (Buscaglia et al., 2008). A recent meta-analysis concluded that the SBI had limited validity for lesion detection in VCE but fared better in the detection of active gastrointestinal bleeding, supporting its use in the acute setting. (Yung et al., 2017b). In an interesting study by Han et al. (2018) , eight contiguous SBI markers was identified as the optimal threshold for detecting GIB (Figure 2B). In a prospective trial, the threshold of eight contiguous SBI markers had a 100% sensitivity and specificity for the detection of active gastrointestinal bleeding. For now, the SBI remains suboptimal for formal reading, but is a useful screening tool for active GIB in particular when the threshold of eight contiguous markers is used.

**TABLE 4:** Selected studies evaluating the testing characteristics of the SBI. PPV, NPV, and overall accuracy are included where reported.

Author(s)	Published	Indication	Size (*n*)	Sensitivity (%)	Specificity (%)	PPV	NPV	Accuracy
Liangpunsakul	2003	IDA, Abdominal pain	24	25.7	85.0	90.3	14.2	34.8
D'Halluin	2005	Obscure GIB	156	37.0	59.0	50.0	46.0	
Signorelli	2005	Obscure GIB, Crohn’s diseas	95	40.9	70.7	69.2	42.6	l58.9
		Metastatic carcinoid, miscellaneous						
Buscaglia	2008	Anemia, obscure GIB, Crohn’s disease, other	291	56.4	33.5	24.0	67.3	
Yung^a^	2017	GIB	2040	55.3	57.8			

a Meta-analysis of studies.

The emergence of AI has also proliferated additional CNN outside of the SBI for the detection of gastrointestinal bleeding (Table 2). For instance, Leenhardt et al. (2019) developed a CNN to detect one of the most common bleeding lesions in the small bowel: The angioectasia. The AI model was trained on 600 images of typical angioectasias and then tested against a panel of human experts. The CNN demonstrated a high diagnostic performance with a sensitivity of 96%, specificity of 100%, and PPV of 96%. More recent CNNs such as that of Tsuboi et al. (2020) have likewise demonstrated excellent outcomes in angioectasia detection).

AI has also been applied to the evaluation of Crohn’s disease through the creation of CNN that can detect and categorize mucosal inflammation. In 2020, Klang et al. (2020) introduced a CNN trained on 17,640 capsule images of normal mucosa or mucosal ulcers that was able to detect mucosal ulcers with an accuracy of 95.4%–96.7%. The same CNN was later applied to grade the severity of mucosal ulcers and was able to do so with an overall accuracy of 62%–91%, with higher accuracy in discriminating between high versus low-grade ulcers. (Barash et al., 2021). In a similar fashion, a CNN has been applied to the detection of erosions and ulcerations outside of Crohn’s disease with equally impressive testing characteristics. (Aoki et al., 2019).

Tumor detection in VCE is another promising area for the application of VCE. Initial studies first proposed an algorithm that could parse color texture and pattern in order to recognize tumors with an accuracy of 92.4%–95%. (Charisis et al., 2012; Li and Meng, 2012). Multiple groups have proposed various methods of parsing imaging data to facilitate computer-aided diagnosis with VCE. Most methods utilize a combination of color and texture data, abstracted to a neural network or similar enhancement technique, in order to analyze images. (Pan et al., 2011; Xing et al., 2018). For example, Silva and colleagues proposed a classification method that incorporated shape and texture in order to detect polyps with an overall detection rate of 68%. (Silva et al., 2013). More recently, deep learning algorithms have been applied to tumor detection. Yuan et al. developed a deep learning model that was able to discriminate between polyps, bubbles, turbid images, and clear images on capsule endoscopy with an accuracies of 95.5%–99.5% (Yuan and Meng, 2017). In one of the largest studies to date, Saito et al. (2020) trained a CNN on 30,584 images from 292 patients that was then able to detect “polyps, nodules, epithelial tumors, submucosal tumors, and venous structures” with sensitivities between 77% and 95.8%. In a recent systematic review of artificial intelligence in VCE, Kim et al. (2022) evaluated 12 studies related to the use of computer-aided diagnosis (CAD) in the detection of protruding lesions of the small bowel. Of these, most studies (58%) involved an Asian population with relatively few (25%) involving a Western population. The authors found an overall sensitivity and specificity of CAD for the detection of protruding lesions of 89% and 91%, respectively. The positive and negative predictive values were 9.3 and 0.13, also respectively.

Other conditions for which AI has been applied include celiac disease, GIB, and overall abnormal findings (Table 3). In a systematic review of studies evaluating the use of artificial intelligence in VCE, Qin et al. (2022) included 16 articles covering the application of artificial intelligence towards ulcers and erosions, gastrointestinal bleeding, and polyps and cancer. The sensitivity and specificity of the CNN for the detection of pathology was high overall, with the highest sensitivity and specificity for gastrointestinal bleeding at 97% and 100%, respectively. The sensitivity and specificity of the algorithms for the detection of erosions and ulcers was 96% and 97%, and for polyps and tumors was 97% and 98%, also respectively. Zhou et al. (2017) were further able to train a CNN using the GoogleNet architecture to distinguish patients with and without celiac disease with a sensitivity of 100%—albeit on a very small testing set of 5 patients. Aoki et al.(2020), in a separate CNN, was able to detect blood content with a sensitivity, specificity, and accuracy approaching 100%. Lastly, Ding et al. (2019) evaluated the role of a CNN in detecting all-comer abnormalities. The CNN improved lesion detection compared to human readers (99.9% vs. 74.6%). Perhaps more meaningfully, the CNN also drastically reduced the reading time compared to human readers (5.9 vs. 96.6 min; *p* < 0.001). The major advantages of CNN are their potential to reduce reading time, and in doing so, expedite triage towards the most appropriate procedure. This is of particular benefit in cases of gastrointestinal bleeding, where the early identification of, for example, a bleeding angioectasia, can assist the endoscopist in choosing the correct procedure (for example, upper endoscopy versus deep enteroscopy versus colonoscopy) to target the offending lesion. Other advantages relate to optimizing lesion detection through the elimination of human reading at all, which requires specific training and is demanding of both time and attention.

The challenges of AI and CNN relate to the challenges of AI as a whole, as well as challenges specific to VCE. For example, additional studies must be done to reproduce CNN findings on external patient populations and confirm the robustness of the algorithms. Algorithms trained on certain populations, or using certain platforms, might not reproduce the same testing characteristics when applied to different patient populations or tested with images from a different capsule endoscope. In fact, it is expected that the testing characteristics of developed CNN will decrease when applied to outside populations. Another challenge involves the adaptation of artificial intelligence over time. The implementation of artificial intelligence requires a robust team capable of monitoring perform, assessing for undetected biases, and deciding when and how to update the algorithm with new data. In addition, in certain diseases that require endoscopic intervention (active gastrointestinal bleeding) or tissue diagnosis (celiac disease), the utility and cost-effectiveness of VCE, with or without the support of CNN, remains an area of ongoing research.

Technical requirements pose another challenge. Development of a CNN requires large, labelled datasets which have historically been the domain of large academic or research-oriented institutions. Smaller hospitals and practices might not have access to the volume of data needed to create a population-specific CNN. In addition, CNN require advanced computing infrastructure, including graphic processing units, to execute their functions. (Faes et al., 2019). Hospitals without the computational power or technical expertise to develop it will likely rely on outsourced, commercial products for AI-based needs.

### Novel imaging methods

In addition to upgrading existing video capsule technologies through hardware and software, novel methods of wireless endoscopic imaging have also been tested. To-date, VCE has been limited to obtaining surface-level mucosal images. However, new methods of imaging, such as micro ultrasound imaging and fluorescent enhanced imaging, could change this in the near future.

For instance, Qiu et al. (2020) utilized high-frequency (>20 MHz) ultrasound to obtain transmural images of the gastrointestinal tract. The so-called ultrasound capsule endoscopy (USCE) was tested in the intestines of anesthetized pigs and was able to achieve differentiating images of the lumen wall up to a depth of 10 mm. The novel technique has potential for the wireless evaluation of submucosal lesions.

In fluorescent enhanced VCE, another novel form of imaging, fluorescent dye is first injected and then detected using special sensors. (Hakimian et al., 2021a). The technique has been proposed to help in distinguishing old blood within the GI tract from active extravasation during VCE in a similar manner to a conventional angiogram. A related technique, called biochromoendoscopy, relies upon special sensors to detect specific wavelengths of light emitted from malignant or premalignant lesions. In biochromoendoscopy, a synthetic probe is injected into the patient but remains undetectable until it reaches the lesion of interest, where it is acted upon by local tissue factors and releases a near-infrared fluorescence (NIRF) signal that can then be detected by attuned sensors on the capsule endoscope. Zhang et al. (2008) published a proof of principle trial of the biochromoendoscopy technique, in which VCE was able to discriminate between adenomas and benign lesions by detecting the NIRF signal released from a cathepsin-B activated probe. The same cathepsin-activated probe was able to spotlight adenocarcinomas in a gastric cancer murine model. (Ding et al., 2012).

Still more imaging methods are under development. Schostek has described a novel sensor that can detect blood based on optical characteristics alone and discriminate between active bleeding and other liquids, including red-colored liquids. (Schostek et al., 2016). The Check-Cap is a “capsule that emits and detects ultra-low dose radiation [and] generates a 3D reconstruction of the colonic lumen for detection of polyps and cancer.” (Chatrath and Rex, 2014; Kimchy et al., 2017)

Together, advancements in hardware, AI, and novel imaging techniques could untether a new field: optical biopsy. Optical biopsy refers to the technique of obtaining a diagnosis based on imaging characteristics alone. Optical biopsy has demonstrated potential in Barrett’s esophagus and other esophageal pathologies, where a low-cost tethered capsule endoscope was able to capture distinctive images of the GEJ in a proof of concept trial. (Seibel et al., 2008; Gora et al., 2016). With continued improvements in camera hardware and the advent of new smart networks with which to interpret captured images, more and more gastrointestinal pathologies, such as Barrett’s dysplasia and gastric metaplasia, will be able to be diagnosed with imaging alone. The Compact Photonics Explorer (CPE), for example, is a remote wireless device comprised of a multitude of sensing applications. (Wang et al., 2005).

In cases where “tissue remains the issue,” VCE has obstacles left to traverse. First and foremost, is the need for consistent navigation and positional holding to allow for precise sampling. Nevertheless, tissue acquisition remains a research interest within VCE and there is hope on the horizon. Several “full-stack” devices are in development that incorporate a full suite of imaging and diagnostic capabilities. The European VECTOR project, for example, is one such device that integrates various diagnostic and therapeutic functionalities into a video capsule. (Schostek and Schurr, 2013). In another example, Ye et al. (2022) described a wireless capsule endoscope that contained a biopsy needle that could be “sprung out” to sample tissue. The capsule was able to collect 0.35 mm^3^ of tissue per sample.

## Advancements in indication

Conventional endoscopy has remained a staple of the gastroenterologist’s toolbox since it became a clinical reality in the 1970s. Yet, as with all technologies, conventional endoscopy is vulnerable to change. It is possible that in the next few decades VCE will start to replace conventional endoscopy as the diagnostic standard for lumenal pathologies. Significant technological hurdles remain, but the promise of a non-invasive, no-sedation needed endoscopic tool represents a motivating result.

In their current form, video capsules transit the entire GI tract, opening new avenues for diagnosis. Current evidence-based indications for VCE include suspected small bowel bleeding and Crohn’s disease. More recent potential indications for VCE include acute GIB, esophagitis, portal hypertensive bleeding including esophageal varices and portal hypertensive gastropathy, and colorectal polyps.

### Gastrointestinal bleeding

The management of acute GIB has remained much the same over the past five decades excepting a number of therapeutic enhancements. Current guidelines recommend resuscitation and risk stratification, followed by the conventional endoscopy of choice. (Laine et al., 2021). VCE introduces a new tool to the evaluation of acute GIB with promising initial results in terms of both risk stratification and subsequent diagnosis.

In one randomized clinical trial of patients with hematemesis, the early use of VCE allowed for the early discharge of up to 80% of patients from the emergency department (ED). (Sung et al., 2016). The discharged patients underwent upper endoscopy in the next few days without any loss of safety when compared with the conventional approach. Similar observations were made by Meltzer et al. (2021) in a truncated multicenter trial.

In another study evaluating the role of capsule endoscopy in the diagnosis of suspected diverticular bleeding, small bowel bleeding was detected in 12% of patients. Additional lesions with high bleeding potential and colonic bleeding were detected in 57% and 12% of patients. However, major clinical outcomes including rates of rebleeding and mortality were unchanged, and so the authors concluded “routine CE is unnecessary for presumptive CDB patients after colonoscopy.” (Aoki et al., 2022)

In one study evaluating the use of VCE in acute non-hematemesis GIB, VCE was demonstrated to be twice as effective as conventional endoscopy in the detection of the site of active bleeding. In the first randomized clinical trial, the use of the Olympus-EC-10 capsule captured more than 60% of the sources of non-hematemesis GIB compared to conventional endoscopy, which captured approximately 30% of sources. (Marya et al., 2019). In a similar study, the use of capsule endoscopy compared to upper endoscopy in acute upper GIB detected an additional source of bleeding in the small bowel in 18% of cases. (Ching et al., 2019a). Compared to hematemesis GIB, the differential for non-hematemesis GIB is quite broad. Non-hematemesis GIB can originate from the nose to the right colon and as such can be a diagnostic challenge for conventional endoscopy. These patients who are poorly diagnosed with the traditional upper endoscopy and colonoscopy often go on to require additional measures such as VCE, CT-guided or catheter-guided angiography, or in severe cases surgery if the site of bleeding is not detected. This diagnostic sequence can take considerable time, which in turn allows for bleeding to stop spontaneously, leaving the gastroenterologist in a state of ignorance as to the original source of bleeding despite the expenditure of a wide range of resources. The expedited deployment of a video capsule, ideally in the ED or a clinical decision unit (CDU), can optimize the chance of detecting the source of bleeding. Once the gastroenterologist recognizes the source of bleeding, they can either immediately deploy endoscopic treatment or choose to continue the evaluation in the outpatient setting, if appropriate.

VCE also offers benefits for patients who present with GIB in resource-constrained settings or settings at risk for environmental exposure. In a trial using historical controls with propensity matching, VCE was deployed for both hematemesis and non-hematemesis bleeding. The trial was conducted during the COVID-19 pandemic and VCE was chosen for this trial in order to minimize exposure risk because it does not generate aerosols. (Hakimian et al., 2021b). The benefits of a VCE-first approach, as compared to the traditional approach of procedural sedation and conventional endoscopy, included a reduction in staff exposure to the virus, a reduction in the use of personal protective equipment (PPE), and a reduction in the number of unnecessary procedures. The patient’s overall length of stay showed a mild reduction.

### Gastroesophageal reflux disease and non-cardiac chest pain

VCE has also demonstrated potential in discriminating between GERD and functional dyspepsia (FD). In clinical practice, GERD and FD are difficult to distinguish. This failure has contributed to the widespread and often inappropriate use of proton pump inhibitors (PPIs), which have come under increasing criticism for a number of associated adverse effects. (Schnoll-Sussman and Katz, 2017). In order to establish a true diagnosis of GERD, either upper endoscopy or catheter-based pH testing is required. Empiric treatment with a PPI can also be used albeit with suboptimal testing characteristics. (Gyawali and Fass, 2018). VCE has demonstrated success in detecting esophagitis and can do so without the need for sedation. (Eliakim et al., 2005). For example, VCE can detect esophagitis with an accuracy of 100%. (Chen et al., 2019). VCE has been approved by the U.S. Food and Drug Administration (FDA) for this indication and has a much broader potential use in discriminating between GERD and FD. It might also have a potential use in the setting of acute chest pain in the ED. In this setting, an estimated 10% of patients have true acute coronary syndrome (ACS). (Singh et al., 2010). A pilot study of patients who presented to the ED with acute chest pain demonstrated that 40% possessed some form of an esophageal disorder, confirmed with earlier endoscopic data. (Dickman et al., 2007). Thus, of those 90% of patients who present to the ED with acute chest pain and without ACS, it is possible that up to 40% might have a similar esophageal disorder. Diagnosing this population of patients with a specific disorder could in turn lead to continued appropriate treatment and theoretically reduce the high rate of recidivism and re-visits to the ED for non-cardiac chest pain. The application of VCE in such a population has been demonstrated to be simple and safe and could even be cost-effective.

Barrett’s esophagus represents another reflux-related disease that stands to benefit from advancements in VCE. In one study evaluating the role of VCE in screening for Barrett’s esophagus, authors Sami et al. reported that a transnasal capsule endoscope could detect Barrett’s with a sensitivity and specificity of 95% and 87% when compared to conventional endoscopy. The procedure was safe and was preferred by patients. (Sami et al., 2019). For now, despite its improved comfort for patients, capsule endoscopy is not recommended in the evaluation of Barrett’s esophagus because of its inability to obtain biopsies, rendering it not a cost-effective option. (Krishna Chandar et al., 2020).

### Cardiopulmonary conditions

Patients with certain cardiopulmonary conditions comprise a unique, high-risk population in which VCE could be of particular benefit. In patients requiring lung transplantation, for example, conventional endoscopy is extremely hazardous and usually not performed, in part because of the risks of cardiopulmonary complications from anesthesia. In patients requiring lung transplantation and where esophageal injury is suspected, the use of VCE might provide valuable information in the assessment of mucosal injury in this seriously ill patient population.

Another setting in which VCE could be of benefit is as an anesthesia-sparing alternative to trans-esophageal echocardiography (TEE). In the future, it is entirely possible to conceive of a tethered capsule that uses ultrasound rather than white light, which could be used as a replacement for TEE for patients with cardiopulmonary conditions. Nascent experiments into ultrasound capsule endoscopy are ongoing, and if brought to clinical fruition, would represent a much more elegant solution than the current and rather cumbersome endoscopically based technology that requires some form of anesthesia (Hakimian et al., 2021b).

### Cirrhosis and portal hypertensive bleeding

Cirrhosis of the liver is often associated with the development of esophagogastric varices and portal hypertensive changes of the gastric and small intestinal mucosa. The latter is generally not evaluated unless all other sources of bleeding are excluded.

VCE has already demonstrated success in the noninvasive screening of esophageal varices, with an accuracy for the detection of varices approaching 67%. (Wang et al., 2021)^,^ (Deding et al., 2021)^,^ (Chen et al., 2019) In the future, VCE could supplant the need for sedation and upper endoscopy in this very ill patient population. The combination of tethered capsules and magnetic-controlled capsule endoscopy (MCE) could further revolutionize the endoscopic exam in patients with chronic liver disease. For example, one proposed approach would be to start with a tethered capsule for the detection of esophageal varices, immediately followed by MCE using the same capsule to search for the presence of gastric varices, portal hypertensive gastropathy, and portal hypertensive enteropathy. In doing so, one noninvasive test could provide complete information on the state of much of the GI tract in chronic liver disease.

### Colorectal polyps

The colon capsule (Medtronic, Minneapolis, MN) has been proven to be an effective tool for screening for colon cancer albeit with several limitations. The most important limitation being that the capsule is just a diagnostic tool and not a therapeutic one. Therefore, the colon capsule has yet to be reimbursed by most insurance providers outside of a narrow context.

The economic argument against colon capsule endoscopy (CCE) is that the prevalence of polyps is greater than 50%, and as such, at least half the population screened with CCE would go on to require a therapeutic colonoscopy, whereas starting with a colonoscopy would be both diagnostic and therapeutic. However, newer research has suggested that not all small polyps need to be removed. Recent studies have estimated the polyp detection rate up to 70%–80%.^69^In a systematic review, the testing characteristics of CCE increased based on polyp size, reaching a sensitivity and specificity of 79%–96% and 66%–97% for polyps >6 mm, and a sensitivity of 84%–97% for polyps >10 mm. In the same review, the colorectal cancer detection rate was 93%. (Vuik et al., 2021). If technological advancements in AI and optical biopsy are further able to delineate which polyps require resection and which do not, it might be possible to reduce the number of colonoscopies required after CCE, in turn making CCE a more cost-effective procedure. In addition, the other major obstacle to adoption of CCE—the need for the reader to interpret two concurrent video streams—could be ameliorated with the use of AI and machine learning. Mascarenhas et al. (2022) have reported promising results in this area, training a CNN to detect protruding lesions on CCE with an overall accuracy of 95.3%.

## Advancements in navigation

In comparison to advancements in diagnosis, advancements in navigation have just started to approach clinical use and with few exceptions remain in the *ex vivo* stages of research. Current video capsule platforms, in widespread clinical use, transit the gastrointestinal tract by harnessing the digestive tract’s natural peristaltic contractions. This method, termed passive locomotion, minimizes the need for a power-intensive locomotion module. However, in doing so it sacrifices maneuverability. The resulting compromise is a wide variation in capsule transit time, mucosal exposure, and missed lesions. In response, active locomotion, or the direct user-controlled movement of the capsule, has garnered significant research interest because of its potential to allow for more precise capsule navigation.

### Onboard controlled locomotion

Active locomotion can be accomplished through either onboard controlled or externally controlled locomotion. In onboard controlled locomotion, the movement of the capsule is facilitated by an on-board actuator that interfaces with mechanical appendages to effect motion. Several unique designs have been tested although none have thus far progressed to large-scale human trials. Example designs have included inchworm-based, leg-based, paddle-based, flap-based, flagellar-based, and propellor-based models.

In the inchworm-based model, “cyclic compression/extension of shape memory alloy (SMA) actuators and anchoring systems” help the capsule to crawl along the intestinal tract. (Ciuti et al., 2016)^,^ (Kwon et al., 2007) In the leg-based model, a set of legs are actuated thanks to a miniaturized motor. Quirin et al. (2007) have developed both 4 and 8-legged models with several degrees of motion in vitro tests. The leg-based model has been also tested in animal models with speeds up to 92 mm/min. (Menciassi et al., 2006; Park et al., 2006). In a proof of principle trial, Qurini was able to pilot the leg-based capsule in a porcine colon for 5 min and cover a distance of 15 cm against peristalsis. (Quirini et al., 2008). The paddling-based model has likewise been tested in both *ex-vivo* and *in vivo* porcine models. In the living porcine model, the paddling-based capsule was able to achieve velocities up to 17 cm/min without serious complications except for pinpoint erythematous mucosal injuries. (Kim et al., 2010; Yang et al., 2011). More recent descriptions of onboard controlled locomotion have included “rubber vibrating legs,” which “skid” along the mucosal surface. (Khan et al., 2022).

The best studied of the group has been the propellor-based model. Tortora and colleagues introduced a propellor-based model in 2009, consisting of a 15 mm × 30 mm capsule “composed of a supporting shell containing a wireless microcontroller, a battery and four motors.” (Tortora et al., 2009) The rear-situated rotating propellors in turn enabled the steering of the capsule for up to 30 min (Tortora et al., 2009). De Falco et al. (2014) iterated on this model and incorporated a camera module that could be used to navigate the capsule for up to 13 min through a video stream.

Overall, onboard controlled locomotion has been limited by technological constraints related to the burden of incorporating an actuator into capsule, alongside batteries strong enough to power it, and at a scale small enough to be comfortably swallowed.

### Externally controlled locomotion

Externally controlled locomotion, on the other hand, has proven more promising. Externally controlled locomotion uses magnetic fields to interface with on-board magnetic components and direct the movement of the capsule. Externally controlled locomotion does not require integrated actuators or engines and thus reduces the engineering hurdles to obtain active motion. Externally controlled locomotion, sometimes called magnet-capsule endoscopy (MCE) or magnetic-controlled capsule endoscopy (MCCE), can be achieved through the use of either small permanent magnets or larger electromagnets that generate a magnetic field. The latter tend to interface with robot-control stations that deliver improved control and coordination at the cost of additional equipment. Historically, work in the area of MCE started with a collaborative effort between the Olympus Corporation and Siemens using MRI magnets and standard capsules. (Rey et al., 2012). This project demonstrated that the capsule could be moved *in vivo* under video surveillance. However, the amount of ferrous material in a conventional capsule is small and, despite the strength of an MRI-based magnetic field, the ability to move the capsule remained limited. Subsequently, the addition of ferrous material into the capsule’s construction has transformed the field.

Permanent magnets can be small enough to incorporate into handheld devices, which a human operator can use to navigate the capsule. Carpi et al. (2007) described the magnetic control of commercial capsules (M2A, Given Imaging Ltd., Yoqneam, Israel) that had been modified with an elastic shell mixed with magnetic particles. In a first in human trial, the additional camera in a PillCam COLON 2 capsule was replaced with magnets and a handheld magnet was used to control the capsule in the esophagus and stomach for 10 min without discomfort. (Swain et al., 2010). Since then, several studies have evaluated the use of MCE in human volunteers. Keller was able to manipulate a capsule using a handheld magnet in a series of volunteers for an average of 39 min and with inspection of 75% of the gastric mucosa in most subjects. (Keller et al., 2011). In a recent prospective cohort trial performed by Ching et al. (2019b), the remote-controlled capsule was compared to standard gastroscopy in the evaluation of refractory iron-deficiency anemia (IDA). In evaluation of 49 patients, the capsule identified more total lesions (88 vs. 52) and more IDA-associated lesions (20 vs. 10) and was associated with lower scores for pain, discomfort, and distress than conventional gastroscopy. Current commercial devices include the OMOM Controllable Capsule System (Jianshan Science and Technology, Chongqing, China) and the Mirocam Navi (Intromedic Ltd., Seoul, Korea). (Shamsudhin et al., 2017). In the Mirocam-Navi platform, a handheld magnet is used to control a ×11 24-mm capsule with a weight of 4.2 g. The magnet can generate a field up to 0.5 T in strength with a corresponding magnetic force of 268-gram force. In an evaluation of 26 volunteers, the Mirocam-Navi platform was shown to be able to successfully visualize a series of important gastrointestinal landmarks 88%–100% of the time. (Rahman et al., 2016).

Compared to handheld permanent magnets, electromagnets that generate magnetic fields are bulkier and require more equipment but offer superior precision. Carpi et al. (2011) were able to adapt an existing “robotic magnetic navigation system (Niobe, Stereotaxis, Inc., United States) already used for cardiovascular procedures” to VCE. (Carpi and Pappone, 2009). Using the robot, Carpi reported the first demonstrated robotic steering of an endoscopic capsule throughout the esophagus, stomach, small bowel, and colon of an *in vivo* pig model. (Carpi and Pappone, 2009) The benefit of the robot-controlled model has been improved stabilization compared to manual controls. For example, in a comparison between robot-assisted control and manual control, Ciuti found that “in *ex vivo* conditions, robotic-assisted control was superior to manual control in terms of targets reached” (87% vs. 37%). (Ciuti et al., 2010). In a more recent experiment, Arezzo et al. (2013) “[aimed] to evaluate feasibility and accuracy of a novel robotically-driven magnetic capsule for colonoscopy as compared to the traditional technique.” The experiment was performed with eleven experts and eleven trainees, who were instructed to find installed pins placed inside a porcine bowel. Arezzo found high completion rates in both techniques, although the procedure took considerably longer in the capsule group (556 vs. 194 s; *p* = 0.0001). Interestingly, the trainees also performed better than the experts at pin detection (87.6% vs. 74.2%; *p* < 0.0001).

**FIGURE 3:**
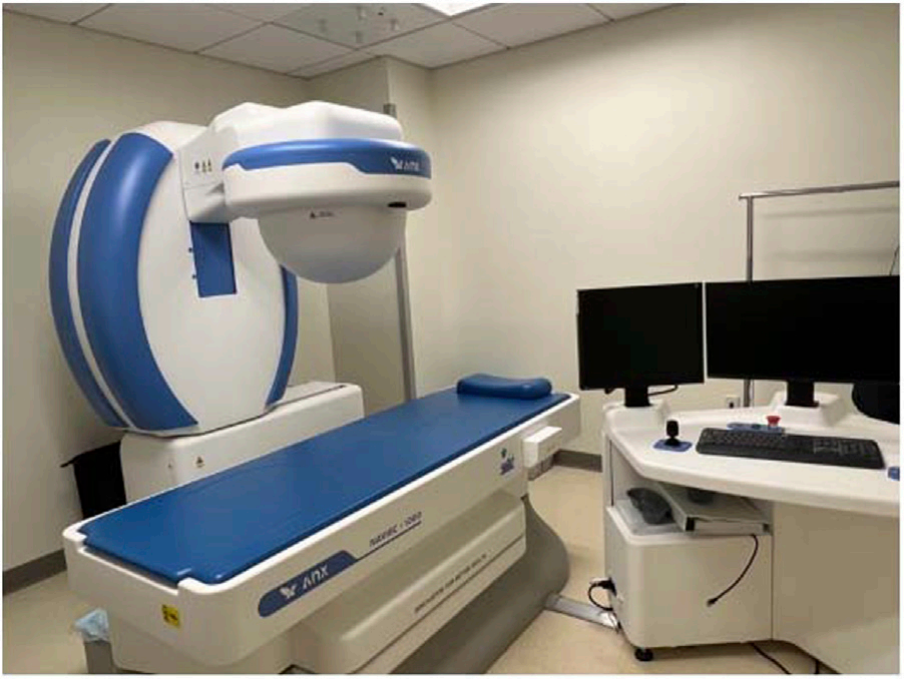
Clinical set-up pf the Navicam (Anx Robotics, Pleasanton:CA) robotic-controlled MCE system. The magnet, housed in the half dome, is controlled through workstation, on the right, that allows the user to manipulate the magnet with respect to a patient, whose has swallowed a capsule and is lying on the bed.

The robot-controlled MCE has reached the point of clinical reality with the Navicam system (ANKON Corp, Wuhan, China; and AnX Robotics, Pleasanton, CA) (Figure 3). In China, the Navicam system has been used extensively as a screening tool in gastric cancer, which remains a major public health issue. In the Navicam protocol, after overnight fasting, the patient drinks about 750 ml of simethicone-containing water and then swallows the endoscopic capsule. During the exam, the patient is placed in various positions under a magnet affixed to a mechanical C-arm to facilitate manual control of the capsule using a pair of remote joysticks. The C-arm has five degrees of freedom and can generate a magnetic field up to 0.2 T across a 50 × 50 × 50 cm^3^ working area. (Shamsudhin et al., 2017). Field strengths between 5 and 30 mT are used to control the capsule, which is monitored under continuous video streaming and its orientation is monitored graphically. (Shamsudhin et al., 2017). Human trials of the Navicam platform have demonstrated promising results. For example, in one study of 34 healthy volunteers, “visualization of the gastric cardia, fundus, body, angulus, antrum, and pylorus was subjectively assessed as complete in 82.4%, 85.3%, 100.0%, 100.0%, 100.0% and 100.0%, respectively.” The total procedure time took 43.8 min (Liao et al., 2012). Expansion of its use using a tether has been reported for demonstrating esophageal varices. (Wang et al., 2021). In addition to promising clinical results, studies have demonstrated improved tolerance using MCE compared to conventional endoscopy. For example, Tai et al. (2022) compared patient tolerance of MCE versus flexible upper endoscopy, and found that patient distress and discomfort both during and after upper endoscopy were significantly increased compared to MCE.

More recent studies of MCE include a prospective, European trial in which 284 patients underwent MCE for examination of the gastric mucosa. To ensure safety, patients with contraindications to VCE, including previous abdominal surgery, previous capsule retention, implanted MRI-incompatible devices or MRI-incompatible foreign bodies, were excluded. The authors further ensured safety by administering a patency capsule beforehand and excluding those with capsule retention. Patients underwent physical exams and were observed throughout the procedure. The overall diagnostic yield was 81.9%, with 74.2% of all abnormalities being detected in the stomach. Diagnostic findings included ulcers, polyps, gastritis, signs of Crohn’s disease, and signs of celiac disease. Challenges of MCE included a prolonged completion time (the average time of the MCE exam was 348 min) and low rate of maneuvering the capsule past the pylorus (41.9%). (Szalai et al., 2022).

On the other hand, Liu et al. (2022) conducted a large-scale trial of MCE of 768 patients across two hospitals in China and demonstrated a much higher rate of capsule passage through the pylorus, at 92.58%. The authors further reported that MCE examined >90% of the gastric mucosa in 94.92% of patients, and completed an entire small bowel examination in 97.40% of patients.

Jiang et al. (2020) compared two versions of MCE in a randomized trial across 80 consecutive patients and demonstrated the second generation capsule improved visualization of the esoophagus and duodenal papilla. The authors also reported a drastically shorter gastric examination completion time, at 5.27 min, likely related to differences in steering and protocol.

Despite the potential of MCE, challenges remain, mostly related to the technological and time investment. In addition to the infrastructure required to perform externally controlled locomotion (Figure 3), more recent reports have confirmed prolonged examination times. (Szalai et al., 2022)^,^ (Kim and Nam, 2021)

## Advancements in localization

VCE poses unique challenges in terms of localization. Unlike upper endoscopy and colonoscopy, which benefit from much shorter lumens and endoscopically identifiable landmarks, VCE must transit the entire small bowel, which is 18–22 feet long and without landmarks. Nevertheless, accurate localization remains crucial to positioning lesions and targeting subsequent invasive therapies.

The major methods of localization in the small intestine include transit-time-based localization, electromagnetic-wave-based localization and magnetic-field-based localization. (Than et al., 2012). The most often used approach remains transit-time-based localization, in which the position of the capsule is estimated based on reader-identified landmarks. (Marya et al., 2014). In this approach, the stomach entrance, duodenum entrance, and cecum entrance are identified, and the capsule position at a point-in-time estimated based on the time elapsed between two landmarks. The technique is not precise but allows for an approximation of capsule location. Given Imaging has also experimented with electromagnetic localization, in which external sensors placed in predefined locations on the patient’s abdomen detect radio frequency (RF) signals emitted from the capsule as it transits the gastrointestinal tract. A “localization algorithm” then predicts the location of the capsule based on the strength of the signal in relation to the external sensors. (Fischer et al., 2004). In an evaluation of the Given Imaging localization algorithm compared against true positions as determined by fluoroscopic images, the average error was found to be 3.77 cm. (Fischer et al., 2004). Furthermore, location was calculated based on distance from the umbilicus and might not reflect true intralumenal location. Consequently, the technique has been withdrawn. Other localization methods that have seen some commercial use include a three-dimensional (3D) localization system developed by Olympus. (Marya et al., 2014). Like with the Given Imaging system, the Olympus system used RF-attenuation system to approximate the location of the wireless capsule. However, unlike the Given Imaging system, the Olympus system also incorporated a Z-dimension to allow for 3D localization. In a validation study involving 30 volunteers, Marya et al. (2014) found that the average error of the 3D system, compared to fluoroscopic controls, to be 2.00, 2.64, and 2.51 cm in the X, Y, and Z dimensional coordinates, respectively. Intromedic has also developed an electric potential value based method. (Ciuti et al., 2016). However, as VCE continues to expand its indication, more precise methods that possess the power to measure distance travelled from the pylorus will be needed in order to optimize diagnostic and therapeutic potential.

Magnetic-field-based localization remains an active area of investigation. (Than et al., 2012). In magnetic-field-based localization, a permanent magnet is incorporated into the capsule and the magnetic flux, as detected by external sensors, is used to determine the capsule’s orientation and location. Permanent magnets have been demonstrated to be accurate to within 4.3% and 2% with regards to distance traveled and orientation. (Zeising et al., 2022). Weitschies et al. (1997) demonstrated it was possible to monitor the transit of a magnetically marked capsule with a spatial resolution in the range of millimeters. Magnetic-field-based localization benefits from being able to pass through human tissue without attenuation and without the use of ionizing radiation. In addition, magnetic-field-based methods appear to be accurate, with localization within the range of 5–7 mm. (Vedaei and Wahid, 2021). However, magnetic-field-based localization is also fragile and prone to interference from other magnetic sources and have yet to be validated in human *in vivo* studies. (Than et al., 2012).

Multiple other methods of localization have been studied. (Than et al., 2012). More novel proposals for localization include that of Chandrappan et al. (2010) who developed a tagging module that releases a bio-compatible micro-tag upon command. The tag embeds into the GI mucosa and can be identified on X-ray, providing localizing information for future procedures. In addition to electromagnetic methods, other research groups have investigated localization methods based on gamma scintigraphy and magnetic resonance. (Ciuti et al., 2016). One interesting innovation is that of the Odocapsule, which contains three wheels that function as odometers and calculates the real-time distance of the capsule from the duodenum. (Karargyris and Koulaouzidis, 2015).

## Conclusion

The expansion of VCE from evaluation of small bowel bleeding and into diagnosing esophageal, colon, and now pan-gastrointestinal tract pathologies would not have been possible without continued technological improvements in imaging and locomotion.

Advancements in imaging have included both hardware and software improvements each coming during a time of exponential engineering advancements. Improvements in camera technologies have led to the development of 2 and 4 camera capsules, while improvements in non-white light imaging have led to the development of flexible spectral imaging color enhancement (FICE), narrow band imaging (NBI) including blue light imaging, and fluorescent enhanced imaging. The next frontier—artificial intelligence and machine learning—has now arrived and promises to expand the reach of VCE even further as the field of optical biopsy becomes a clinical reality.

With advancements in active locomotion increasing, we could soon see the commercial development of universal capsule endoscopes capable of diagnostic and therapeutic capabilities for all manner of indications. For now, VCE has solidified its role in the evaluation of small bowel bleeding and earned an indispensable spot in the practicing gastroenterologist’s armamentarium.
